# Characteristics of Maternal Mortality Missed by Vital Statistics in Hong Kong, 2000-2019

**DOI:** 10.1001/jamanetworkopen.2023.0429

**Published:** 2023-02-22

**Authors:** Ka Wang Cheung, Mimi Tin-Yan Seto, Weilan Wang, Po Lam So, Annie S. Y. Hui, Florrie Nga-Yui Yu, Wai Hang Chung, Wendy Shu, Minnie Yim, Tiffany Sin-Tung Au, Tsz Kin Lo, Ernest Hung Yu Ng

**Affiliations:** 1 Department of Obstetrics and Gynaecology, Queen Mary Hospital, University of Hong Kong, Hong Kong, China; 2 Department of Obstetrics and Gynaecology, Tuen Mun Hospital, Hong Kong, China; 3 Department of Obstetrics and Gynaecology, Prince of Wales Hospital, Hong Kong, China; 4 Department of Obstetrics and Gynaecology, Queen Elizabeth Hospital, Hong Kong, China; 5 Department of Obstetrics and Gynaecology, United Christian Hospital, Hong Kong, China; 6 Department of Obstetrics and Gynaecology, Pamela Youde Nethersole Eastern Hospital, Hong Kong, China; 7 Department of Obstetrics and Gynaecology, Kwong Wah Hospital, Hong Kong, China; 8 Department of Obstetrics and Gynaecology, Princess Margaret Hospital, Hong Kong, China

## Abstract

**Importance:**

Reducing maternal mortality is a global objective. The maternal mortality ratio (MMR) is low in Hong Kong, China, but there has been no local confidential enquiry into maternal death, and underreporting is likely.

**Objective:**

To determine the causes and timing of maternal death in Hong Kong and identify deaths and their causes that were missed by the Hong Kong vital statistics database.

**Design, Setting, and Participants:**

This cross-sectional study was conducted among all 8 public maternity hospitals in Hong Kong. Maternal deaths were identified using prespecified search criteria, including a registered delivery episode between 2000 to 2019 and a registered death episode within 365 days after delivery. Cases as reported by the vital statistics were then compared with the deaths found in the hospital-based cohort. Data were analyzed from June to July 2022.

**Main Outcomes and Measures:**

The outcomes of interest were maternal mortality, defined as death during pregnancy or within 42 days after ending the pregnancy, and late maternal death, defined as death more than 42 days but less than 1 year after end of the pregnancy.

**Results:**

A total of 173 maternal deaths (median [IQR] age at childbirth, 33 [29-36] years) were found, including 74 maternal mortality events (45 direct deaths and 29 indirect deaths) and 99 late maternal deaths. Of 173 maternal deaths, 66 women (38.2%) of individuals had preexisting medical conditions. For maternal mortality, the MMR ranged from 1.63 to 16.78 deaths per 100 000 live births. Suicide was the leading cause of direct death (15 of 45 deaths [33.3%]). Stroke and cancer deaths were the most common causes of indirect death (8 of 29 deaths [27.6%] each). A total of 63 individuals (85.1%) died during the postpartum period. In the theme-based approach analysis, the leading causes of death were suicide (15 of 74 deaths [20.3%]) and hypertensive disorders (10 of 74 deaths [13.5%]). The vital statistics in Hong Kong missed 67 maternal mortality events (90.5%). All suicides and amniotic fluid embolisms, 90.0% of hypertensive disorders, 50.0% of obstetric hemorrhages, and 96.6% of indirect deaths were missed by the vital statistics. The late maternal death ratio ranged from 0 to 16.36 deaths per 100 000 live births. The leading causes of late maternal death were cancer (40 of 99 deaths [40.4%]) and suicide (22 of 99 deaths [22.2%]).

**Conclusions and Relevance:**

In this cross-sectional study of maternal mortality in Hong Kong, suicide and hypertensive disorder were the dominant causes of death. The current vital statistics methods were unable to capture most of the maternal mortality events found in this hospital-based cohort. Adding a pregnancy checkbox to death certificates and setting up a confidential enquiry into maternal death could be possible solutions to reveal the hidden deaths.

## Key Points

**Question:**

How well did the vital statistics database capture the incidence and causes of maternal mortality in Hong Kong?

**Findings:**

In this cross-sectional study including 173 maternal deaths from 2000 to 2019, 91% of maternal mortality events identified were missed by the vital statistics. The leading cause of maternal mortality was suicide, and all suicide and amniotic fluid embolism deaths, 90% of hypertensive disorder deaths, 50% of obstetric hemorrhage deaths, and 97% of indirect deaths were missed by the vital statistics.

**Meaning:**

These findings suggest that the vital statistics database did not reflect the true incidence of maternal mortality in Hong Kong, which could misguide allocation of resources.

## Teaser

This cross-sectional study assesses causes and timing of maternal death in Hong Kong and identifies deaths and their causes that were missed by the vital statistics database.

## Introduction

Maternal mortality is defined as the death of a pregnant individual while pregnant or within 42 days of the termination of the pregnancy from any cause related to or aggravated by the pregnancy or its management but not from accidental causes.^1^ The global maternal mortality ratio (MMR), defined as the number of maternal mortality events per 100 000 live births, remains unacceptably high.^2,3^ The United Nations Maternal Mortality Estimation Interagency Group^2^ reported a 38% reduction in the MMR, from 342 to 211 maternal deaths per 100 000 live births, between 2000 and 2017, with approximately 295 000 maternal deaths in 2017. Acceleration is necessary to meet the ambitious target of an MMR less than 70 per 100 000 live births by 2030, set by the Sustainable Development Goals of the United Nations.^3^

Much attention was focused on low- and middle-income countries (LMICs) for contributing a significant burden to the global MMR.^4^ Although the MMR among high-income countries (HICs) reduced from 13 to 11 deaths per 100 000 live births between 2000 and 2017, a notable increase was observed in the US in the past decade.^2,5^ In HICs, a potential surge in maternal mortality despite modern obstetric care could be due to the changing demographics of pregnant individuals, for example, delaying childbearing so that a greater proportion of individuals were in advanced maternal age, used assisted reproductive techniques, or had complex medical conditions when contemplating pregnancy.^6^ The causes of maternal death also differed between LMICs and HICs; obstetric hemorrhage was the leading cause among LMICs, while indirect maternal death and thromboembolism were the dominant causes in HICs.^7,8,9^

Based on the data from vital statistics, we previously found a very low MMR in Hong Kong, declining from 125 deaths per 100 000 live births in 1946 to 1.8 deaths per 100 000 live births in 2017. Thromboembolism (37.0% of deaths) and obstetric hemorrhage (30.4% of deaths) were identified as the principal causes of maternal mortality in Hong Kong, while sepsis and indirect maternal death were rare.^10^ An in-depth evaluation of each maternal death using information from the vital statistics was not feasible but could be elucidative to inform policy makers on investments in targeted preventive health care strategies. Furthermore, pregnant individuals who committed suicide were frequently not counted in the vital statistics of Hong Kong.^11^ There was no confidential enquiry into maternal death in Hong Kong. We hypothesized that data on maternal mortality events were potentially underreported to the vital statistics database. Therefore, we aimed to evaluate the causes of all maternal deaths in all public hospitals in Hong Kong between 2000 to 2019 and compare that with maternal deaths reported in the vital statistics.

## Methods

We carried out a multicenter cross-sectional study involving all 8 public maternity hospitals in Hong Kong (Kwong Wah Hospital, Pamela Youde Nethersole Eastern Hospital, Prince of Wales Hospital, Princess Margaret Hospital, Queen Elizabeth Hospital, Queen Mary Hospital, Tuen Mun Hospital, and United Christian Hospital). Ethical approval was obtained from the institutional review board of each unit, and a waiver of informed consent was granted because this was a retrospective study. This study is reported following the Strengthening the Reporting of Observational Studies in Epidemiology (STROBE) reporting guideline.

The hospital-based data were obtained via the Clinical Data Analysis and Reporting System. Cases were identified using the following search criteria: a registered delivery episode between January 1, 2000, to December 31 2019, and a registered death episode within 365 days after delivery. A retrospective health record review was performed, and clinical details were retrieved from the computerized clinical database (Clinical Management System; Hospital Authority). Basic demographics and clinical details were collected, including the date of delivery, date and location of maternal death, gravida, parity, age, educational level, marital status, ethnicity, smoking and alcohol consumption history, underlying medical conditions, gestational age at delivery, mode of delivery, and the outcome of the fetus. Reviews of paper medical records were performed for missing data. Individuals with accidental death (defined as unnatural death due to accident) or incomplete data were excluded.

Outcomes were defined similar to the World Health Organization’s recommendation.^1^ Maternal mortality was defined as maternal death from any cause related to or aggravated by pregnancy or its management (excluding accidental or incidental causes) during pregnancy and childbirth or within 42 days of the end of the pregnancy, irrespective of the duration and site of the pregnancy. Maternal mortality was further classified into direct and indirect maternal death. Direct maternal death was defined as death resulting from obstetric complications of the pregnant state (pregnancy, labor, and puerperium) and from interventions, omissions, incorrect treatment, or from a chain of events resulting from any of the above. Indirect maternal death was defined as death resulting from previous existing disease or disease that developed during pregnancy and not due to direct obstetric causes but was aggravated by the physiologic effects of pregnancy. Late maternal death was defined as the death of an individual within more than 42 days but less than 1 year of the end of the pregnancy.

Timing of maternal mortality was divided into antepartum (from conception to the onset of labor), intrapartum (during labor and delivery) and postpartum death (after and within 42 days of delivery). The causes of maternal mortality were classified using *International Classification of Diseases–Maternal Mortality* (*ICD-MM*), which divided causes of death into 9 main categories under direct and indirect maternal death.^12^ Subclassification was performed for common causes of death in Hong Kong: other obstetric complications of direct maternal death included suicide, amniotic fluid embolism, cardiac diseases, thromboembolism, and others; indirect maternal death included infection (except HIV and hepatitis B infection), cardiac diseases, stroke, hepatitis B infection, cancer, and others. Late maternal death included suicide, cancer, cardiac diseases, stroke, infection (except HIV and hepatitis B infection), HIV, thromboembolism, others, and unknown. We also adopted a theme-based approach to analyze the cause of maternal mortality events and categorized them into suicide, cancer-related death, infection (including HIV and hepatitis B infection), stroke, cardiac diseases, obstetric hemorrhage, amniotic fluid embolism, thromboembolism, hypertensive disorders, and others.^13^

For the population-based cohort, the numbers of maternal mortality per year between 2000 and 2019 were extracted from the vital statistics database by identifying the number of registered maternal deaths with the cause of death classified as a cause under chapter XV “Pregnancy, childbirth and the puerperium” of the *International Statistical Classification of Diseases and Related Health Problems, Tenth Revision (ICD-10)*, as provided by the Department of Health, the Government of the Hong Kong Special Administrative Region.^10^ Vital statistics of Hong Kong were not intended to capture late maternal death; therefore, population-based data on late maternal death were not available. For maternal mortality, direct matching of each case between the hospital-based cohort and the population-based cohort was not feasible due to a lack of personal information reported by the vital statistics; therefore, we performed approximate matching. Each death with an identical cause that occurred within the same year was assumed to be the same case, based on the rationale of a very low maternal mortality in Hong Kong.

### Statistical Analysis

Categorical data were presented as number and percentage, while for numerical data, median values with IQRs were used. The differences of demographic or clinical characteristics between the direct and indirect maternal death were investigated by *t* test for normally distributed continuous variables, Mann-Whitney test for skewed continuous variables, and χ^2^ test or Fisher exact test for categorical variables. For neonatal outcomes, differences between the outcomes from an antepartum or intrapartum and postpartum maternal death were investigated by *t* test or Mann-Whitney test for continuous variables or Fisher exact test for categorical variables. Data analysis was performed on R statistical software version 4.1.0 (R Project for Statistical Computing, and visualization was performed using Excel version 16.68 (Microsoft). A two-sided *P* value <0.05 was considered statistically significant. Data were analyzed from June to July 2022

## Results

A total of 181 maternal deaths and 1 280 354 live births were recorded within the study period. Eight patients were excluded, 5 due to accidental death and 3 for missing data, leaving 173 for final analysis. Table 1 shows the basic demographics of all individuals. There were 74 maternal mortality events (45 direct and 29 indirect) and 99 late maternal deaths. The median (IQR) maternal age was 33 (29-36) years; 172 individuals (99.4%) were Asian and 1 individual was South American. Of 173 maternal mortality events, 66 (38.2%) were among individuals with preexisting medical conditions.

**Table 1.** Basic Demographics Among All Maternal Deaths During Pregnancy or Within 1 Year of the End of Pregnancy

Characteristic	No. (%) (N = 173)
Maternal age at estimated date of childbirth, mean (SD), y	33 (29-36)
Gestational age at delivery, mean (SD), wk	38 (34.4-39.4)
Nulligravida	57 (33.3)
Nulliparity	93 (53.8)
Marital status	
Married	141 (81.5)
Other	32 (18.5)
Missing	0
Educational level	
≤Secondary	107 (61.8)
≥Tertiary	31 (17.9)
Missing	35 (20.2)
Smoking	
Active	19 (11.0)
Former smoker	14 (8.1)
Nonsmoker	98 (56.6)
Missing	42 (24.3)
Drinking	
Active	14 (8.1)
Former drinker	8 (4.6)
Nondrinker	102 (59.0)
Missing	49 (28.3)
Location of death	
Inpatient	117 (67.6)
Outside hospital	56 (32.4)
Mode of conception	
Spontaneous	161 (93.1)
Assisted reproductive technique	9 (5.2)
Missing	3 (1.7)
Preexisting medical condition	
Drug abuse	11 (6.4)
Asthma	7 (4.0)
Psychiatric disorder	13 (7.5)
Hypertension	4 (2.3)
Neurological disorder	3 (1.7)
Cardiac disease	5 (2.9)
Malignant neoplasm	8 (4.6)
Kidney disease	3 (1.7)
Diabetes	2 (1.2)
Syphilis	1 (0.6)
Thyroid disease	4 (2.3)
Hepatitis B carrier	11 (6.4)
Hemoglobinopathy	4 (2.3)
Polycystic ovarian syndrome	2 (1.2)
Fibroid	3 (1.7)
Others	1 (0.6)
None	107 (61.8)
Multiple pregnancy	7 (4.0)
Classification of death	
Direct	45 (26.0)
Indirect	29 (16.8)
Late	99 (57.2)
Timing of death	
Antepartum death	9 (5.2)
Gestational age of maternal death, wk	32.7 (23.6-35.2)
Intrapartum death	2 (1.2)
Gestational age of maternal death, wk	36.5 (34.0-39.0)
Postpartum death	162 (93.6)
Days from delivery, d	38 (12-266)

The Figure showsthe triennial trend of all death ratios during pregnancy or within 1 year after end of pregnancy. eTable 1 in Supplement 1 lists the annual numbers and the death ratios per 100 000 live births of direct, indirect, and late maternal death between 2000 and 2019.

**Figure.**
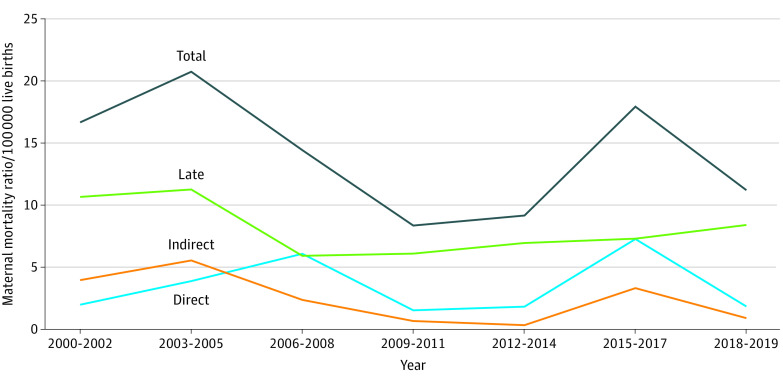
The Triennial Women’s Mortality Ratio During Pregnancy or Within 1 Year of the End of Pregnancy Maternal mortality was defined as death during pregnancy or within 42 days after ending the pregnancy, and was further classified into direct and indirect maternal death. Direct maternal death was defined as death resulting from obstetric complications of the pregnant state (pregnancy, labor, and puerperium) and from interventions, omissions, incorrect treatment, or from a chain of events resulting from any of these. Indirect maternal death was defined as death resulting from previous existing disease or disease that developed during pregnancy and not due to direct obstetric causes but was aggravated by the physiologic effects of pregnancy. Late maternal death was defined as death more than 42 days but less than 1 year after end of the pregnancy.

### Maternal Mortality

The MMR from 2000 to 2019 ranged from 1.63 to 16.78 deaths per 100 000 live births. Direct death accounted for 60.8% of maternal mortality, while 39.2% of individuals died from indirect causes. No significant trend was observed in the numbers of total, direct, or indirect deaths during the studied period.

Table 2 provides the triennial number and causes of total, direct and indirect maternal mortality events from 2000 to 2019 (yearly breakdowns are in eTable 2 and eTable 3 in Supplement 1). Of 45 direct deaths, suicide was the leading cause (15 deaths [33.3%]), followed by hypertensive disorders (10 deaths [22.2%]) and obstetric hemorrhage (8 deaths [17.8%]). Of 29 indirect deaths, stroke and cancer the most common causes, with 8 deaths (27.6%) each, followed by cardiac diseases (4 deaths [13.8%]).

**Table 2.** Total, Direct, and Indirect Maternal Mortality Events by Cause From 2000 to 2019

	Total, No. (%)	Deaths, No.						
		2000-2002	2003-2005	2006-2008	2009-2011	2012-2014	2015-2017	2018-2019
Total	74	9	14	18	6	5	19	3
Direct deaths								
Total	45 (100)	3	6	13	4	4	13	2
Pregnancies with abortive outcome	1 (2.2)	0	0	0	0	0	1	0
Hypertensive disorders in pregnancy, childbirth, and the puerperium	10 (22.2)	0	0	5	1	1	3	0
Obstetric hemorrhage	8 (17.8)	1	3	1	0	1	2	0
Pregnancy-related infection	2 (4.4)	0	0	0	0	0	1	1
Suicide	15 (33.3)	2	2	4	1	0	6	0
Amniotic fluid embolism	6 (13.3)	0	0	2	1	2	0	1
Cardiac diseases	1 (2.2)	0	1	0	0	0	0	0
Pulmonary embolism	1 (2.2)	0	0	1	0	0	0	0
Others obstetric complications	1 (2.2)	0	0	0	1	0	0	0
Indirect deaths								
Total	29 (100)	6	8	5	2	1	6	1
Infection (exclude HIV and hepatitis B)	3 (10.3)	0	2	1	0	0	0	0
Cardiac diseases	4 (13.8)	0	4	0	0	0	0	0
Stroke	8 (27.6)	3	1	0	2	0	1	1
Hepatitis B infection	3 (10.3)	0	1	2	0	0	0	0
Cancer-related death	8 (27.6)	2	0	1	0	1	4	0
Others	3 (10.3)	1	0	1	0	0	1	0

The sociodemographics and location of mortality between individuals with direct and indirect death are shown in eTable 4 in Supplement 1. There were no significant differences except for more out-of-hospital maternal mortality among direct deaths than indirect deaths in the postpartum period (12 individuals [33.3%] vs 2 individuals [7.4%]; *P* = .02). Reclassifying individuals with suicide to indirect death reversed the observation (0 individuals vs 14 individuals [35.0%]; *P* < .001) (eTable 5 in Supplement 1). Most direct (excluding suicide) and indirect deaths happened within the hospital (29 direct deaths [96.7%] and 26 indirect deaths [89.7%]), compared to only 1 death (6.7%) among individuals who died of suicide. The median (IQR) delivery-to-death duration of the postpartum death cases varied among direct (2 [1-6] days), indirect death (13 [5-21] days), and suicide deaths (14 [9-28] days) (eTable 6 in Supplement 1).

There were 9 antepartum deaths (12.2%), 2 intrapartum deaths (2.7%) and 63 postpartum deaths (85.1%) (Table 3). Among 9 antepartum deaths, 3 individuals died of hypertensive disorders, 2 individuals died of suicide, and 1 individual died of a cardiac event, stroke, amniotic fluid embolism, or other cause. Intrapartum deaths were caused by hypertensive disorder in 1 individual and amniotic fluid embolism in the other individual. Regarding postpartum deaths, 5 individuals (7.9%), died on the day of delivery, 30 individuals (47.6%) died 1 to 10 days after delivery, and 28 individuals (44.4%) died 11 to 42 days after delivery (Table 3).

**Table 3.** Timing of Maternal Deaths in Relation to Pregnancy

Timing	No. (%)		*P* value	Total (N = 74), No. (%)
	Direct death (n = 45)	Indirect death (n = 29)		
Antenatal and intrapartum period	9 (20.0)	2 (6.9)	.18	11 (14.9)
Postnatal period, d from delivery				
On day of delivery	4 (8.9)	1 (3.4)	.38	5 (6.8)
1-5	16 (35.6)	6 (20.7)	.11	22 (29.7)
6-10	6 (13.3)	2 (6.9)	.45	8 (10.8)
11-20	4 (8.9)	9 (31.0)	.06	13 (17.6)
21-42	6 (13.3)	9 (31.0)	.15	15 (20.3)

eTable 7 in Supplement 1 presents the mode of delivery and the neonatal outcomes of maternal mortality events. There were 14 fetuses among 11 antepartum or intrapartum deaths (3 sets of twins) and 67 fetuses among 63 postpartum deaths (2 sets of twins and 1 set of triplets). Perimortem cesarean delivery was performed on 6 pregnant individuals (1 twin pregnancy); all were antepartum deaths except 1 individual who committed suicide and had successful resuscitation but died on day 3 after delivery and so was classified as postpartum death. The stillbirth rate was higher among antepartum or intrapartum than postpartum death (6 individuals [42.9%] vs 5 individuals [7.5%]; *P* = .003) and fewer newborns were still alive until hospital discharge among individuals with antepartum or intrapartum death (7 newborns [50.0%] vs 56 newborns [83.6%], *P* = .01). There were no significant differences in other neonatal outcomes. eTable 8 and eTable 9 in Supplement 1 present the possible risk factors associated with maternal death (antepartum or intrapartum vs postpartum; direct vs indirect death). There were no significant differences in the different ranges of maternal age, preexisting medical conditions, history of psychiatric conditions, and the number of antenatal visits.

In the theme-based approach analysis of 74 deaths, the leading causes of mortality, in descending order, were suicide (15 deaths [20.3%]); hypertensive disorders (10 deaths [13.5%]); obstetric hemorrhage, infection, stroke, or cancer (8 deaths [10.8%] each); amniotic fluid embolism (6 deaths [8.1%]); cardiac disease and others (5 deaths [6.8%] each) and thromboembolism (1 death [1.4%]). eTable 10 in Supplement 1 shows the basic demographics, timing, and location of maternal mortality among different themes of maternal mortality. Of note, among individuals who died of suicide, 3 (21.4%) were active smokers or active drinkers, and 3 (20.0%) had preexisting psychiatric disorders. In addition, 3 individuals (37.5%) who died of infection-related causes had hepatitis B.

The vital statistics in Hong Kong only recorded 26 maternal mortality events between 2000 and 2019, and comparison of the causes of death with our hospital-based cohort is shown in Table 4. Only 7 deaths were found to overlap with the deaths recorded in our hospital-based cohort. The vital statistics database failed to capture 67 maternal deaths in our cohort, while we could not identify 16 cases listed in the vital statistics after excluding 3 deaths with unknown causes. All suicides and amniotic fluid embolisms, 90.0% hypertensive disorders (9 deaths), 50.0% of obstetric hemorrhages (4 deaths), and 96.6% of indirect deaths (28 deaths) were missed by the vital statistics. Regarding timing of maternal mortality, 8 antepartum deaths (88.9%), 2 intrapartum deaths (100%), and 57 postpartum deaths (90.5%) were not reported to vital statistics. On the other hand, our cohort could not identify most of the deaths caused by thromboembolism and early pregnancy complications (eg, abortion or ectopic pregnancy).

**Table 4.** Comparison of the Causes of Death Between Vital Statistics and Hospital-Based Data

Year	Deaths, No.		Deaths, No. (cause of death)				
	Vital statistics	Hospital-based data	Common cases	Missed by vital statistics		Missed by hospital-based data	
				Indirect	Direct	Indirect	Direct
2000	3	3	1 (obstetric hemorrhage: postpartum death on day of delivery)	2 (2 cancer)	0	0	2 (1 hypertensive disorder; 1 abortion-related)
2001	1	2	1 (indirect maternal death: postpartum death on day 3)	1 (stroke)	0	0	0
2002	1	4	0	2 (1 stroke; 1 other)	2 (2 suicide)	0	1 (Thromboembolism)
2003	2 (1 unknown cause)	8	1 (obstetric hemorrhage: postpartum death on day 1)	7 (2 infections; 4 cardiac conditions; 1 stroke)	0	0	0
2004	2 (one unknown cause)	4	0	1 (infection)	3 (1 cardiac condition; 1 obstetric hemorrhage; 1 suicide)	0	1 (thromboembolism)
2005	2	2	1 (obstetric hemorrhage: postpartum death on day 1)	0	1 (suicide)	0	1 (abortion-related)
2006	1	5	0	1 (infection)	4 (1 amniotic fluid embolism; 2 hypertensive disorder; 1 suicide)	0	1 (thromboembolism)
2007	1	9	0	4 (2 infection; 1 cancer; 1 other)	5 (1 amniotic fluid embolism; 1 hypertensive disorders; 1 obstetric hemorrhage; 2 suicide)	0	1 (thromboembolism)
2008	2	4	1 (thromboembolism: postpartum death on day 9)	0	3 (2 hypertensive disorder; 1 suicide)	0	1 (obstetric hemorrhage)
2009	2	2	0	0	2 (1 amniotic fluid embolism; 1 other)	0	2 (obstetric hemorrhage)
2010	1	2	0	0	2 (1 hypertensive disorder; 1 suicide)	0	1 (thromboembolism)
2011	1	2	0	2 (2 stroke)	0	0	1 (obstetric hemorrhage)
2012	2	3	1 (obstetric hemorrhage: postpartum death on day 1)	1 (cancer)	1 (hypertensive disorder	0	1 (ectopic pregnancy)
2013	0	1	0	0	1 (amniotic fluid embolism)	0	0
2014	2 (one unknown cause)	1	0	0	1 (amniotic fluid embolism)	0	1 (thromboembolism)
2015	1	8	0	3 (2 cancer; 1 other)	5 (2 hypertensive disorder; 1 infection; 2 suicides	0	1 (ectopic pregnancy)
2016	0	6	0	2 (1 stroke; 1 cancer)	4 (1 abortion-related; 2 obstetric hemorrhage; 1 suicide)	0	0
2017	1	5	1 (hypertensive disorder: antepartum death at 31 gestational wk)	1 (cancer)	3 (3 suicide)	0	0
2018	1	2	0	1 (Stroke)	1 (amniotic fluid embolism)	0	1 Thromboembolism
2019	0	1	0	0	1 (infection)	0	0
Total	26	74	7	28 (8 cancer; 6 infection; 7 stroke; 4 cardiac disease; 3 other)	39 (15 suicide; 9 hypertensive disorder; 6 amniotic fluid embolism; 4 obstetric hemorrhage; 2 infection; 1 cardiac condition; 1 other; 1 abortion-related)	0	16 (7 thromboembolism; 4 obstetric hemorrhage; 2 abortion-related; 2 ectopic pregnancy; 1 hypertensive disorder)

### Late Maternal Death

The late maternal death ratio ranged from 0 to 16.36 deaths per 100 000 live births (eTable 1 in Supplement 1). eTable 11 in Supplement 1 presents the annual numbers and causes of late maternal death per year between 2000 and 2019. Of 99 late maternal deaths, the leading causes were cancer (40 deaths [40.4%]), suicide (22 deaths [22.2%]), cardiac diseases (12 deaths [12.1%]), infection (7 deaths [7.1%]), unknown (7 deaths [7.1%]), stroke (5 deaths [5.1%]), others (3 deaths [3.0%]), HIV-related (2 deaths [2.0%]), and thromboembolism (1 death [1.0%]). No significant trends were observed in numbers and causes of late maternal deaths.

## Discussion

This cross-sectional study found that the Hong Kong vital statistics database failed to capture 90.5% of maternal mortality events from public hospitals in Hong Kong, most of the missed deaths being suicides, amniotic fluid embolism, hypertensive disorders, and indirect deaths. This finding suggests that a review in the reporting mechanism of maternal mortality is required. Both direct and indirect maternal deaths contributed significantly to maternal mortality events during pregnancy and immediately after delivery in Hong Kong. There was no significant decrease in direct, indirect, or late maternal death nor temporal changes in the cause of death, likely due to the low number of maternal mortality events during the studied period. The leading cause of death was suicide, and the theme-based approach showed that the other common causes of maternal death were hypertensive disorder, obstetric hemorrhage, infection, stroke, and cancer. Most maternal deaths were in the postpartum period, and only 2.7% of deaths were intrapartum deaths. A significant number of individuals died between 42 days and 1 year after delivery and were not recorded by vital statistics. Suicide and cancer continued to be the major causes of late maternal death.

Previous reports on maternal mortality in Hong Kong were based on vital statistics. Between 1961 and 1985, despite a significant decline in the number of maternal mortality events, obstetric hemorrhage and preeclampsia accounted for approximately 34% and 20% of maternal mortality, respectively, in Hong Kong.^14^ Between 1986 and 1990, pulmonary embolism became the primary cause of death (53% of deaths) after the 1986 implementation of compulsory coroner postmortem examination for all maternal deaths improved detection.^15^ It was alarming to note the significant differences between our cohort and a 2022 report on the analysis of maternal mortality in Hong Kong.^10^ We found that indirect deaths were common and that there were fewer deaths from thromboembolism than previously reported. Mortality statistics could miss up to 81% of indirect deaths.^16^ Thromboembolism may not be a major cause of maternal mortality in a predominately Chinese population: in another Hong Kong study,^17^ the incidence of thromboembolism during pregnancy was 0.4 per 1000 pregnancies, which was lower than the reported risk of 1 to 2 events per 1000 pregnancies in predominantly White populations, such as in the United Kingdom.^18^ Four cases of thromboembolism in the vital statistics and amniotic fluid embolism in our cohort were reported within the same year (2006, 2007, 2014, and 2018), which was possibly due to incorrect coding, since amniotic fluid embolism had not previously been coded as a cause of death in the vital statistics. Inaccurate coding may misguide allocation of resources, since the approaches to prevent maternal death from thromboembolism and amniotic fluid embolism are completely different.

Despite finding missing maternal deaths, the overall incidence of maternal mortality in Hong Kong remains low, and the reasons are likely multifactorial and have been previously described.^10^ Hong Kong is densely populated, with an efficient transportation network. The government funds health care services to ensure universal coverage, and no one is denied access to medical care due to a lack of means. Policy-directed health programs at a low cost optimize baseline health at a population level. Pregnant individuals are tightly engaged with a free, comprehensive, and well-organized antenatal program, enabling earlier identification of high-risk pregnancies and timely management in a multidisciplinary manner, even in minority populations (eg, ethnic minority or individuals with a language barrier). Emergency obstetric services and high-quality intrapartum care and interventions are readily available and further safeguard pregnant individuals and their fetuses. For example, a prompt perimortem cesarean delivery could enhance maternal resuscitation and improve maternal and neonatal survival. Individuals who collapse from severe conditions or who may die during pregnancy or delivery could be resuscitated and delivered speedily but die after delivery. This may partly explain the higher proportion of postpartum death in Hong Kong.

### Limitations

This study has several limitations. First, deaths were only included if both a delivery and a death were registered in public units. The actual number of maternal deaths may be underestimated and would explain the discrepancy between our cohort and the vital statistics. Second, direct case matching with the vital statistics was not possible, potentially affecting the comparison. Nonetheless, our aim was to bring attention to the hidden burden of underreported maternal deaths and emphasize on the need of a better reporting system (eg, death due to amniotic fluid embolism or thromboembolism). Third, assignment into direct or indirect death were difficult for suicide and cancer-related deaths. We adopted a theme-based approach in the analysis to overcome this. Fourth, this cohort may miss deaths in early pregnancy or after elective or spontaneous abortion, as these deaths may not be coded as deliveries in the hospital system.

## Conclusions

This cross-sectional study found that direct and indirect maternal deaths contributed significantly to maternal mortality data in Hong Kong. Suicide and hypertensive disorders were the leading causes of maternal mortality. We found that the current vital statistics did not capture the actual prevalence of maternal death in Hong Kong, most likely due to underreporting (eg, unawareness of recent pregnancy), misunderstanding (eg, unaware of the need to report), misclassification (eg, wrong coding or incorrect identification of underlying pathology), or misinterpretation (eg, believing the death was not related to pregnancy). Adding a pregnancy checkbox to death certificates could facilitate case identification, but proper education and individual case reviews are crucial to avoid a falsely inflated mortality rate.^19,20,21^ The next step would be setting up a confidential enquiry into maternal death, allowing in-depth analyses to study whether and the extent to which a death is associated with pregnancy and identify pitfalls in the health care system and make evidence-based suggestions for prevention.^22^ The perceived lower incidence of maternal mortality from the vital statistics may hamper the evolution of obstetric care, which is common in HICs not using a pregnancy checkbox on death certificates or without a confidential enquiry into maternal death.^23,24^ In addition, many individuals who had given birth died after 42 days and within a year after delivery, which was not captured by the vital statistics. Late maternal death was often a neglected area and was not included in regular statistics.^25^ Suicide, cardiac diseases, and cancer continued to pose heavy burden to late maternal death. To target late maternal death, health care strategies similar to those used during pregnancy and puerperium should be extended to cover the period after delivery.

Pregnancy is a unique life occasion. A well-structured, systematic platform could broadly provide opportunistic health care and education, initiating engagement with existing health services and improving health literacy and awareness, even after delivery and especially for the minority populations. It would likely enhance individual health by modifying the risk factors for noncommunicable disease in the long term at a global level. With our findings of these previously unrecorded maternal deaths now revealed, policy makers and the stakeholders should listen, invest, and find solutions to maintain low maternal mortality with the highest quality of perinatal care.

## Notes

Supplement 1. **eTable 1.** Annual Number of Maternal Deaths and Death Ratio From 2000 to 2019 **eTable 2.** Annual Number and Causes of Direct Maternal Deaths From 2000 to 2019 **eTable 3.** Annual Number and Causes of Indirect Maternal Death From 2000 to 2019 **eTable 4.** Comparison of Basic Demographics and Location of Maternal Mortality Between Direct Death (With Suicide) and Indirect Death **eTable 5.** Comparison of Basic Demographics and Location of Maternal Mortality Between Direct and Indirect Death (With Suicide) **eTable 6.** Comparison of Timing and Location of Maternal Mortality Among Direct, Indirect Cause and Suicide **eTable 7.** Mode of Delivery and Neonatal Outcomes Among Antepartum/ Intrapartum and Postpartum Mortality **eTable 8.** Risk Factors of Maternal Mortality Between Antepartum and Intrapartum vs Postpartum Death **eTable 9.** Risk Factors of Maternal Mortality Between Direct and Indirect Death **eTable 10.** Basic Demographics, Timing and the Location of Maternal Mortality Among Different Themes of Maternal Mortality **eTable 11.** Annual Numbers and Causes of Late Maternal Death From 2000 to 2019

Supplement 2.
Data Sharing Statement
