# Supplementary material for: Characteristics of Maternal Mortality Missed by Vital Statistics in Hong Kong, 2000-2019
Source: JAMA Netw Open. 2023 Feb 22;6(2):e230429. doi: 10.1001/jamanetworkopen.2023.0429 (PMC9947727; doi:10.1001/jamanetworkopen.2023.0429)
Supplement: Supplement 1. — eTable 1. Annual Number of Maternal Deaths and Death Ratio From 2000 to 2019 eTable 2. Annual Number and Causes of Direct Maternal Deaths From 2000 to 2019 eTable 3. Annual Number and Causes of Indirect Maternal Death From 2000 to 2019 eTable 4. Comparison of Basic Demographics and Location of Maternal Mortality Between Direct Death (With Suicide) and Indirect Death eTable 5. Comparison of Basic Demographics and Location of Maternal Mortality Between Direct and Indirect Death (With Suicide) eTable 6. Comparison of Timing and Location of Maternal Mortality Among Direct, Indirect Cause and Suicide eTable 7. Mode of Delivery and Neonatal Outcomes Among Antepartum/ Intrapartum and Postpartum Mortality eTable 8. Risk Factors of Maternal Mortality Between Antepartum and Intrapartum vs Postpartum Death eTable 9. Risk Factors of Maternal Mortality Between Direct and Indirect Death eTable 10. Basic Demographics, Timing and the Location of Maternal Mortality Among Different Themes of Maternal Mortality eTable 11. Annual Numbers and Causes of Late Maternal Death From 2000 to 2019 [file jamanetwopen-e230429-s001.pdf]

Supplemental Online Content

Cheung KW, Seto MTY, Wang W, et al. Characteristics of maternal mortality missed by vital statistics in Hong Kong, 2000-2019. *JAMA Netw Open*. 2023;6(2):e230429. doi:10.1001/jamanetworkopen.2023.0429

eTable 1. Annual Number of Maternal Deaths and Death Ratio From 2000 to 2019
eTable 2. Annual Number and Causes of Direct Maternal Deaths From 2000 to 2019
eTable 3. Annual Number and Causes of Indirect Maternal Death From 2000 to 2019
eTable 4. Comparison of Basic Demographics and Location of Maternal Mortality Between Direct Death (With Suicide) and Indirect Death
eTable 5. Comparison of Basic Demographics and Location of Maternal Mortality Between Direct and Indirect Death (With Suicide)
eTable 6. Comparison of Timing and Location of Maternal Mortality Among Direct, Indirect Cause and Suicide
eTable 7. Mode of Delivery and Neonatal Outcomes Among Antepartum/Intrapartum and Postpartum Mortality
eTable 8. Risk Factors of Maternal Mortality Between Antepartum and Intrapartum vs Postpartum Death
eTable 9. Risk Factors of Maternal Mortality Between Direct and Indirect Death
eTable 10. Basic Demographics, Timing and the Location of Maternal Mortality Among Different Themes of Maternal Mortality
eTable 11. Annual Numbers and Causes of Late Maternal Death From 2000 to 2019

This supplemental material has been provided by the authors to give readers additional information about their work.

© 2023 Cheung KW et al. *JAMA Network Open.*

**eTable 1.** Annual Number of Maternal Deaths and Death Ratio From 2000 to 2019							
Year	Live births	Direct	direct (per 100,000 livebirths)	Indirect	indirect (per 100,000 livebirths)	Late	late (per 100,000 livebirths)
2000	53720	1	1.86	2	3.72	4	7.45
2001	49144	0	0	2	4.07	7	14.24
2002	48119	2	4.16	2	4.16	5	10.39
2003	47687	1	2.10	7	14.68	5	10.49
2004	48914	3	6.13	1	2.04	8	16.36
2005	57124	2	3.50	0	0	4	7.00
2006	65195	4	6.14	1	1.53	5	7.67
2007	70394	5	7.10	4	5.68	0	0
2008	78751	4	5.08	0	0	8	10.16
2009	82906	2	2.41	0	0	9	10.86
2010	88200	2	2.27	0	0	2	2.27
2011	95348	0	0	2	2.10	5	5.24
2012	91343	2	2.19	1	1.09	7	7.66
2013	57623	1	1.74	0	0	2	3.47
2014	61290	1	1.63	0	0	6	9.79
2015	60803	5	8.22	3	4.93	6	9.87
2016	60331	4	6.63	2	3.32	2	3.32
2017	56890	4	7.03	1	1.76	5	8.79
2018	53716	1	1.86	1	1.86	6	11.17
2019	52856	1	1.89	0	0	3	5.68

© 2023 Cheung KW et al. *JAMA Network Open.*

**eTable 2.** Annual Number and Causes of Direct Maternal Deaths From 2000 to 2019										
Year	Direct	Pregnancies with abortive outcome	Hypertensive disorders in pregnancy, childbirth, and the puerperium	Obstetric hemorrhage	Pregnancy-related infection	Suicide	Amniotic fluid embolism	Cardiac diseases	Pulmonary embolism	Others obstetric complications
2000	1	0	0	1	0	0	0	0	0	0
2001	0	0	0	0	0	0	0	0	0	0
2002	2	0	0	0	0	2	0	0	0	0
2003	1	0	0	1	0	0	0	0	0	0
2004	3	0	0	1	0	1	0	1	0	0
2005	2	0	0	1	0	1	0	0	0	0
2006	4	0	2	0	0	1	1	0	0	0
2007	5	0	1	1	0	2	1	0	0	0
2008	4	0	2	0	0	1	0	0	1	0
2009	2	0	0	0	0	0	1	0	0	1
2010	2	0	1	0	0	1	0	0	0	0
2011	0	0	0	0	0	0	0	0	0	0
2012	2	0	1	1	0	0	0	0	0	0
2013	1	0	0	0	0	0	1	0	0	0
2014	1	0	0	0	0	0	1	0	0	0
2015	5	0	2	0	1	2	0	0	0	0

© 2023 Cheung KW et al. *JAMA Network Open.*

Year	Direct	Pregnancies with abortive outcome	Hypertensive disorders in pregnancy, childbirth, and the puerperium	Obstetric hemorrhage	Pregnancy-related infection	Suicide	Amniotic fluid embolism	Cardiac diseases	Pulmonary embolism	Others obstetric complications
2016	4	1	0	2	0	1	0	0	0	0
2017	4	0	1	0	0	3	0	0	0	0
2018	1	0	0	0	0	0	1	0	0	0
2019	1	0	0	0	1	0	0	0	0	0
Total	45	1	10	8	2	15	6	1	1	1

© 2023 Cheung KW et al. *JAMA Network Open.*

**eTable 3.** Annual Number and Causes of Indirect Maternal Death From 2000 to 2019							
Year	Indirect	Infection (exclude HIV and hepatitis B)	Cardiac diseases	Stroke	Hepatitis B infection	Cancer-related death	Others
2000	2	0	0	0	0	2	0
2001	2	0	0	2	0	0	0
2002	2	0	0	1	0	0	1
2003	7	2	4	1	0	0	0
2004	1	0	0	0	1	0	0
2005	0	0	0	0	0	0	0
2006	1	0	0	0	1	0	0
2007	4	1	0	0	1	1	1
2008	0	0	0	0	0	0	0
2009	0	0	0	0	0	0	0
2010	0	0	0	0	0	0	0
2011	2	0	0	2	0	0	0
2012	1	0	0	0	0	1	0
2013	0	0	0	0	0	0	0
2014	0	0	0	0	0	0	0
2015	3	0	0	0	0	2	1
2016	2	0	0	1	0	1	0
2017	1	0	0	0	0	1	0
2018	1	0	0	1	0	0	0
2019	0	0	0	0	0	0	0
Total	29	3	4	8	3	8	3

© 2023 Cheung KW et al. *JAMA Network Open.*

**eTable 4.** Comparison of Basic Demographics and Location of Maternal Mortality Between Direct Death (With Suicide) and Indirect Death			
	Direct death (n=45)	Indirect death (n=29)	P value†
Maternal age at estimated date of confinement (year)	33 (30 – 37)	33 (30 – 36)	0.58
Gestational age at delivery (week)	37.2 (31.3 – 39.1)	36 (32.4 – 38.0)	0.85
Nulligravida	21 (46.7%)	8 (27.6%)	0.20
Nulliparity	31 (68.9%)	14 (48.3%)	0.17
Chinese ethnicity	41 (91.1%)	29 (100%)	0.15
Multiple pregnancy	5 (11.1%)	1 (3.4%)	0.39
Marital status			0.47
• Married	39 (86.7%)	27 (93.1%)	
• Other	6 (13.3%)	2 (6.9%)	
Educational level			1.00
• Secondary or lower	24 (70.6%)	14 (73.7%)	
• Tertiary or higher	10 (29.4%)	5 (26.3%)	
Smoking			0.22
• Active smoker	3 (8.8%)	0 (0%)	
• Ex-smoker	4 (11.8%)	0 (0%)	
• Non-smoker	27 (79.4%)	16 (100%)	
Drinking			0.58
• Active drinker	3 (9.1%)	0 (0%)	
• Ex-drinker	2 (6.1%)	0 (0%)	
• Non-drinker	28 (84.8%)	13 (100%)	
Mode of conception			1.00
• Spontaneous	42 (95.5%)	26 (96.3%)	
• Assisted reproductive technique	2 (4.5%)	1 (3.4%)	
Women with pre-existing medical illness			
• Drug abuse	1 (2.2%)	1 (3.4)	1.00
• Asthma	2 (4.4%)	1 (3.4%)	1.00
• Psychiatric Disease	4 (8.9%)	0 (0%)	0.15
• Hypertension	2 (4.4%)	0 (0%)	0.52
• Neurological disease	1 (2.2%)	0 (0%)	1.00
• Cardiac disease	0 (0%)	2 (6.9%)	0.15
• Malignancy	0 (0%)	1 (3.4%)	0.39
• Renal disease	0 (0%)	1 (3.4%)	0.39

© 2023 Cheung KW et al. *JAMA Network Open.*

	Direct death (n=45)	Indirect death (n=29)	P value†
• Diabetes mellitus	0 (0%)	0 (0%)	NA
• Syphilis	1 (2.2%)	0 (0%)	1.00
• Thyroid disease	1 (2.2%)	0 (0%)	1.00
• Hepatitis B carrier	1 (2.2%)	4 (13.8%)	0.07
• Hemoglobinopathy	0 (0%)	0 (0%)	NA
• Polycystic ovarian syndrome	2 (4.4%)	0 (0%)	0.52
• Fibroid	0 (0%)	0 (0%)	NA
• Others	0 (0%)	0 (0%)	NA
• Nil	33 (73.3%)	21 (72.4%)	1.00
Antepartum death	7 (15.6%)	2 (6.9%)	0.47
● Gestational age at delivery (week)	27.5 (22.9 – 33.6)	35.9 (35.2 – 36.7)	0.09
● Location of death (outpatient)	3 (42.9%)	1 (50.0%)	1.0
Intrapartum death	2 (4.4%)	0 (0%)	0.52
● Gestational age at delivery (week)	36.5 (34.0 – 39.0)	-	NA
● Location of death (outpatient)	0 (0%)	-	NA
Postpartum death (≤ 42 days)	36 (80.0%)	27 (93.1%)	0.18
● Days after delivery (day)	4 (1 – 11)	13 (4.5 – 21)	0.07
● Location of death (outpatient)	12 (33.3%)	2 (7.4%)	0.02
Location of death (outpatient) (Total)	15 (33.3%)	3 (10.3%)	0.03

†p value was calculated by t-test for maternal age, gestational age and days after delivery, and Chi-square test or Fisher’s exact test for the others

© 2023 Cheung KW et al. *JAMA Network Open.*

**eTable 5.** Comparison of Basic Demographics and Location of Maternal Mortality Between Direct and Indirect Death (With Suicide)			
	Direct death (n=30)	Indirect death (n=44)	P value†
Maternal age at estimated date of confinement (year)	34.0 (30.2 – 38.8)	32.5 (29.8 – 36.0)	0.42
Gestational age at delivery (week)	37 (30.6 – 38.3)	36 (33.2 – 38.4)	0.29
Nulligravida	12 (40.0%)	17 (38.6%)	1.00
Nulliparity	20 (66.7%)	25 (56.8%)	0.63
Ethnicity (Chinese)	26 (86.7%)	44 (100%)	0.02
Multiple pregnancy	4 (13.3%)	2 (4.5%)	0.22
Marital status			1.00
• Married	27 (90.0%)	39 (88.6%)	
• Other	3 (10.0%)	5 (11.4%)	
Educational level			0.22
• Secondary or lower	14 (60.9%)	24 (80.0%)	
• Tertiary or higher	9 (39.1%)	6 (20.0%)	
Smoking			0.48
• Active smoker	0 (0%)	3 (10.0%)	
• Ex-smoker	2 (10.0%)	2 (6.7%)	
• Non-smoker	18 (90.0%)	25 (83.3%)	
Drinking			0.10
• Active drinker	0 (0%)	3 (11.1%)	
• Ex-drinker	2 (10.5%)	0 (0%)	
• Non-drinker	17 (89.5%)	24 (88.9%)	
Mode of conception			0.56
• Spontaneous	27 (93.1%)	41 (97.6%)	
• Assisted reproductive technique	2 (6.9%)	1 (2.4%)	
Women with pre-existing medical illness			
• Drug abuse	0 (0%)	2 (4.5%)	0.51
• Asthma	1 (3.3%)	2 (4.5%)	1.00
• Psychiatric Disease	1 (3.3%)	3 (6.8%)	0.64
• Hypertension	1 (3.3%)	1 (2.3%)	1.00
• Neurological disease	1 (3.3%)	0 (0%)	0.41
• Cardiac disease	0 (0%)	2 (4.5%)	0.51
• Malignancy	0 (0%)	1 (2.3%)	1.00
• Renal disease	0 (0%)	1 (2.3%)	1.00
• Diabetes mellitus	0 (0%)	0 (0%)	NA

© 2023 Cheung KW et al. *JAMA Network Open.*

	Direct death (n=30)	Indirect death (n=44)	P value†
• Syphilis	0 (0%)	1 (2.3%)	1.00
• Thyroid disease	1 (3.3%)	0 (0%)	0.41
• Hepatitis B carrier	0 (0%)	5 (11.4%)	0.08
• Hemoglobinopathy	0 (0%)	0 (0%)	NA
• Polycystic ovarian syndrome	2 (6.7%)	0 (0%)	0.16
• Fibroid	0 (0%)	0 (0%)	NA
• Others	0 (0%)	0 (0%)	NA
• Nil	23 (76.7%)	31 (70.5%)	0.75
Antepartum death	5 (16.7%)	4 (9.1%)	0.47
● Gestational age at delivery (week)	31.0 (22.6 – 34.4)	34.4 (29.2 – 35.9)	0.47
● Location of death (outpatient)	1 (20.0%)	3 (75%)	0.21
Intrapartum death	2 (6.7%)	0 (0%)	0.16
● Gestational age at delivery (week)	36.5 (34.0 – 39.0)	-	NA
● Location of death (outpatient)	0 (0%)	-	NA
Postpartum death (≤ 42 days)	23 (76.7%)	40 (90.9%)	0.11
● Days after delivery (day)	2 (1 – 6)	13 (5.8 – 21.2)	<0.001
● Location of death (outpatient)	0 (0%)	14 (35.0%)	<0.001
Location of death (outpatient) (Total)	1 (3.3%)	17 (38.6%)	<0.001

†p value was calculated by t-test for maternal age, gestational age and days after delivery, and Chi-square test or Fisher's exact test for the others.

© 2023 Cheung KW et al. *JAMA Network Open.*

eTable 6. Comparison of Timing and Location of Maternal Mortality Among Direct, Indirect Cause and Suicide

	Direct (n=30)	Indirect (n= 29)	Suicide (n = 15)
Antepartum death	5 (16.7%)	2 (6.9%)	2 (13.3%)
● Gestational age	31 (22.6 – 34.4)	35.9 (35.2 – 36.7)	24 (24 - 24)
● Location of death (outpatient)	1 (20.0%)	1 (50.0%)	2 (100.0%)
Intrapartum death	2 (6.7%)	0 (0%)	0 (0%)
● Gestational age	36.5 (34.0 – 39.0)	-	-
● Location of death (outpatient)	0 (0%)	-	-
Postpartum death (≤ 42 days)	23 (76.7%)	27 (93.1%)	13 (86.7%)
● Days after delivery	2 (1 – 6)	13 (4.5 – 21)	14 (9 – 28)
● Location of death (outpatient)	0 (0%)	2 (7.4%)	12 (92.3%)
Location of death (outpatient) (Total)	1 (3.3%)	3 (10.3%)	14 (93.3%)

© 2023 Cheung KW et al. *JAMA Network Open.*

**eTable 7.** Mode of Delivery and Neonatal Outcomes Among Antepartum/ Intrapartum and Postpartum Mortality			
	Antepartum and intrapartum deaths (n = 14)	Postpartum deaths (n = 67)	P value
Mode of delivery			<0.001
● Not delivered	2 (14.3%)	0 (0%)	0.03
● Normal vaginal delivery	4 (28.6%)	25 (37.3%)	0.01
● Instrumental delivery	0 (0%)	6 (9.0%)	0.58
● Elective lower segment Caesarean section	0 (0%)	5 (7.5%)	0.58
● Emergency lower segment Caesarean section	1 (7.1%)	30 (44.8%)	0.08
● Perimortem Caesarean section	6 (42.9%)	1 (1.5%)	<0.001
● Miscarriage	1 (7.1%)	0 (0%)	0.17
Neonatal outcome			
● Birth weight (grams)	1830 (1471 - 2895)	2512 (1629 - 3102)	0.39
● Apgar score at one minute	6 (3.5 – 8.5)	8 (5 – 9)	0.60
● Apgar score at five minutes	9 (6 – 9.5)	9 (8 – 10)	0.71
● Miscarriage	1 (7.1%)	1 (1.5%)	0.32
● Stillbirth	6 (42.9%)	5 (7.5%)	0.003
● Neonatal death	0 (0%)	5 (7.5%)	0.58
● Alive until hospital discharge	7 (50.0%)	56 (83.6%)	0.01
● Live birth without complications	6 (42.9%)	40 (59.7%)	0.37

† p value was calculated by Mann-Whitney test for birth weight and Apgar score, and Fisher's exact test for others.

© 2023 Cheung KW et al. *JAMA Network Open.*

eTable 8. Risk Factors of Maternal Mortality Between Antepartum and Intrapartum vs Postpartum Death

	Antepartum and intrapartum death (n = 11)	Postpartum death (n = 63)	P value†
Age			0.21
● <20	1 (9.1%)	0 (0%)	
● 20-24	1 (9.1%)	4 (6.3%)	
● 25-29	0 (0%)	12 (19.0%)	
● 30-34	5 (45.5%)	23 (36.5%)	
● 35-39	3 (27.3%)	15 (23.8%)	
● ≥ 40	1 (9.1%)	9 (14.3%)	
With any pre-existing medical illness	7 (63.6%)	32 (50.8%)	0.53
With underlying psychiatric disease	0 (0%)	4 (6.3%)	1.00
Number of antenatal visits			0.17
● <4	6 (60.0%)	19 (34.5%)	
● ≥ 4	4 (40.0%)	36 (65.5%)	

† p value was calculated by Fisher's exact test.

**eTable 9.** Risk Factors of Maternal Mortality Between Direct and Indirect Death			
	Direct death (n = 45)	Indirect death (n = 29)	P value†
Age			0.92
● <20	1 (2.2%)	0 (0%)	
● 20-24	3 (6.7%)	2 (6.9%)	
● 25-29	7 (15.6%)	5 (17.2%)	
● 30-34	15 (33.3%)	13 (44.8%)	
● 35-39	12 (26.7%)	6 (20.7%)	
● ≥ 40	7 (15.6%)	3 (10.3%)	
With any pre-existing medical illness	21 (46.7%)	18 (62.1%)	0.24
With underlying psychiatric disease	4 (8.9%)	0 (0%)	0.15
Number of antenatal visits			0.37
● <4	12 (32.4%)	13 (46.4%)	
● ≥ 4	25 (67.6%)	15 (53.6%)	

† p value was calculated by Chi-square test or Fisher's exact test.

**eTable 10.** Basic Demographics, Timing and the Location of Maternal Mortality Among Different Themes of Maternal Mortality									
	Suicide	Hypertensive disorders	Obstetric hemorrhage	Infection	Stroke	Cancer-related death	Amniotic fluid embolism	Cardiac disease	Others (with thromboembolism)
n	15	10	8	8	8	8	6	5	6
Direct death	15 (100%)	10 (100%)	8 (100%)	2 (25%)	0 (0%)	0 (0%)	6 (100%)	1 (20%)	3 (50%)
Indirect death	0 (0%)	0 (0%)	0 (0%)	6 (75%)	8 (100%)	8 (100%)	0 (0%)	4 (80%)	3 (50%)
Maternal age at estimated date of confinement (year)	31 (29.5 – 35.5)	37 (30.2 – 39.8)	35.5 (33 – 38.5)	32 (26.5 – 34.5)	32 (30.5 – 35.8)	33.5 (31.8 – 34.5)	30.5 (25.5 – 32.5)	30 (23 - 34)	36 (34.5 – 38.2)
Gestational age at delivery (week)	38.4 (33.9 – 39.4)	34.2 (31.1 – 38.2)	37.7 (37 – 38.6)	35.2 (31.8 – 37.6)	38.2 (34.9 – 39.1)	30.5 (27.8 – 32.2)	34.7 (30.7 – 39.9)	37.4 (34 – 37.4)	36.2 (29.2 – 36.9)
Nulligravida	9 (60%)	3 (30%)	2 (25%)	4 (50%)	1 (12.5%)	2 (25%)	4 (66.7%)	2 (40%)	2 (33.3%)
Nulliparity	11 (73.3%)	7 (70%)	3 (37.5%)	5 (62.5%)	3 (37.5%)	4 (50%)	5 (83.3%)	3 (60%)	4 (66.7%)
Ethnicity (Chinese)	15 (100%)	10 (100%)	7 (87.5%)	8 (100%)	8 (100%)	8 (100%)	4 (66.7%)	5 (100%)	5 (83.3%)
Multiple pregnancy	1 (6.7%)	2 (20%)	0 (0%)	1 (12.5%)	0 (0%)	0 (0%)	1 (16.7%)	0 (0%)	1 (16.7%)
Marital status									
• Married	12 (80%)	8 (80%)	8 (100%)	8 (100%)	7 (87.5%)	8 (100%)	5 (83.3%)	4 (80%)	6 (100%)
• Other	3 (20%)	2 (20%)	0 (0%)	0 (0%)	1 (12.5%)	0 (0%)	1 (16.7%)	1 (20%)	0 (0%)
Educational level									
• Secondary or lower	10 (90.9%)	3 (60%)	5 (83.3%)	4 (80%)	6 (100%)	3 (42.9%)	3 (50%)	1 (50%)	3 (60%)

© 2023 Cheung KW et al. *JAMA Network Open.*

• Tertiary or higher	1 (9.1%)	2 (40%)	1 (16.7%)	1 (20%)	0 (0%)	4 (57.1%)	3 (50%)	1 (50%)	2 (40%)
	Suicide	Hypertensive disorders	Obstetric hemorrhage	Infection	Stroke	Cancer-related death	Amniotic fluid embolism	Cardiac disease	Others (with thromboembolism)
Smoking									
• Active smoker	3 (21.4%)	0 (0%)	0 (0%)	0 (0%)	0 (0%)	0 (0%)	0 (0%)	0 (0%)	0 (0%)
• Ex-smoker	2 (14.3%)	1 (14.3%)	0 (0%)	0 (0%)	0 (0%)	0 (0%)	0 (0%)	1 (25%)	0 (0%)
• Non-smoker	9 (64.3%)	6 (85.7%)	6 (100%)	3 (100%)	6 (100%)	4 (100%)	4 (100%)	3 (75%)	2 (100%)
Drinking									
• Active drinker	3 (21.4%)	0 (0%)	0 (0%)	0 (0%)	0 (0%)	0 (0%)	0 (0%)	0 (0%)	0 (0%)
• Ex-drinker	0 (0%)	1 (16.7%)	0 (0%)	0 (0%)	0 (0%)	0 (0%)	0 (0%)	1 (25%)	0 (0%)
• Non-drinker	11 (78.6%)	5 (83.3%)	6 (100%)	2 (100%)	5 (100%)	3 (100%)	4 (100%)	3 (75%)	2 (100%)
Mode of conception									
• Spontaneous	15 (100%)	8 (88.9%)	8 (100%)	6 (85.7%)	8 (100%)	7 (100%)	6 (100%)	5 (100%)	5 (83.3%)
• Assisted reproductive technique	0 (0%)	1 (11.1%)	0 (0%)	1 (14.3%)	0 (0%)	0 (0%)	0 (0%)	0 (0%)	1 (16.7%)
Women with pre-existing medical illness									
• Drug abuse	1 (6.7%)	0 (0%)	0 (0%)	0 (0%)	0 (0%)	0 (0%)	0 (0%)	0 (0%)	1 (16.7%)
	Suicide	Hypertensive	Obstetric	Infection	Stroke	Cancer-	Amniotic	Cardiac	Others (with

© 2023 Cheung KW et al. *JAMA Network Open.*

		disorders	hemorrhage			related death	fluid embolism	disease	thromboembolism)
● Asthma	1 (6.7%)	0 (0%)	0 (0%)	0 (0%)	0 (0%)	0 (0%)	0 (0%)	1 (20%)	1 (16.7%)
● Psychiatric disease	3 (20%)	0 (0%)	1 (12.5%)	0 (0%)	0 (0%)	0 (0%)	0 (0%)	0 (0%)	0 (0%)
● Hypertension	1 (6.7%)	1 (10%)	0 (0%)	0 (0%)	0 (0%)	0 (0%)	0 (0%)	0 (0%)	0 (0%)
● Neurological disease	0 (0%)	1 (10%)	0 (0%)	0 (0%)	0 (0%)	0 (0%)	0 (0%)	0 (0%)	0 (0%)
● Cardiac disease	0 (0%)	0 (0%)	0 (0%)	0 (0%)	0 (0%)	0 (0%)	0 (0%)	2 (40%)	0 (0%)
● Malignancy	0 (0%)	0 (0%)	0 (0%)	0 (0%)	0 (0%)	1 (12.5%)	0 (0%)	0 (0%)	0 (0%)
● Renal disease	0 (0%)	0 (0%)	0 (0%)	0 (0%)	0 (0%)	0 (0%)	0 (0%)	1 (20%)	0 (0%)
● Diabetes melittus	0 (0%)	0 (0%)	0 (0%)	0 (0%)	0 (0%)	0 (0%)	0 (0%)	0 (0%)	0 (0%)
● Syphilis	1 (6.7%)	0 (0%)	0 (0%)	0 (0%)	0 (0%)	0 (0%)	0 (0%)	0 (0%)	0 (0%)
● Thyroid disease	0 (0%)	1 (10%)	0 (0%)	0 (0%)	0 (0%)	0 (0%)	0 (0%)	0 (0%)	0 (0%)
● Hepatitis B carrier	1 (6.7%)	0 (0%)	0 (0%)	3 (37.5%)	0 (0%)	0 (0%)	0 (0%)	1 (20%)	0 (0%)
● Hemoglobinopathy	0 (0%)	0 (0%)	0 (0%)	0 (0%)	0 (0%)	0 (0%)	0 (0%)	0 (0%)	0 (0%)
● Polycystic ovarian syndrome	0 (0%)	1 (10%)	0 (0%)	0 (0%)	0 (0%)	0 (0%)	0 (0%)	0 (0%)	1 (16.7%)
● Fibroid	0 (0%)	0 (0%)	0 (0%)	0 (0%)	0 (0%)	0 (0%)	0 (0%)	0 (0%)	0 (0%)
● Others	0 (0%)	0 (0%)	0 (0%)	0 (0%)	0 (0%)	0 (0%)	0 (0%)	0 (0%)	0 (0%)
● Nil	10 (67.7)	6 (60.0%)	7 87.5%)	5 (62.5%)	8 (100%)	7 (87.5%)	6 (100%)	1 (20.0%)	4 (66.7%)
	Suicide	Hypertensive	Obstetric	Infection	Stroke	Cancer-	Amniotic	Cardiac	Others (with

© 2023 Cheung KW et al. *JAMA Network Open.*

		disorders	hemorrhage			related death	fluid embolism	disease	thromboembolism)
Antepartum death	2 (13.3%)	3 (30%)	0 (0%)	0 (0%)	1 (12.5%)	0 (0%)	1 (16.7%)	1 (20%)	1 (16.7%)
● Gestational age	24 (24 – 24)	31 (26.8 - 32.7)	NA	NA	34.4 (34.4 – 34.4)	NA	38 (38 – 38)	37.4 (37.4 – 37.4)	7 (7 – 7)
● Location of death (outpatient)	2 (100%)	1 (33.3%)	NA	NA	1 (100%)	NA	0 (0%)	0 (0%)	0 (0%)
Intrapartum death	0 (0%)	1 (10%)	0 (0%)	0 (0%)	0 (0%)	0 (0%)	1 (16.7%)	0 (0%)	0 (0%)
● Gestational age	-	41.6 (41.6 – 41.6)	NA	NA	NA	NA	31.4 (31.4 – 31.4)	NA	NA
● Location of death (outpatient)	-	0 (0%)	NA	NA	NA	NA	0 (0%)	NA	NA
Postpartum death (≤ 42 days)	13 (86.7%)	6 (60%)	8 (100%)	8 (100%)	7 (87.5%)	8 (100%)	4 (66.7%)	4 (80%)	5 (83.3%)
● Days after delivery	14 (9 – 28)	5 (1.3 – 8.3)	1 (0.8 – 2.3)	12.5 (9.3 – 14.5)	12 (3 – 16.5)	20 (5 – 21)	1.5 (0 – 5)	29.5 (14.5 – 40.2)	8 (2 – 9)
• Location of death (outpatient)	12 (92.3%)	0 (0%)	0 (0%)	0 (0%)	0 (0%)	0 (0%)	0 (0%)	0 (0%)	2 (40%)
Location of death (outpatient) (Total)	14 (93.3%)	1 (10%)	0 (0%)	0 (0%)	1 (12.5%)	0 (0%)	0 (0%)	0 (0%)	2 (33.3%)

© 2023 Cheung KW et al. *JAMA Network Open.*

**eTable 11.** Annual Numbers and Causes of Late Maternal Death From 2000 to 2019										
Year	Late maternal death	Suicide	Cancer-related death	Cardiac diseases	Stroke	Infection (except HIV and hepatitis B)	HIV	Pulmonary embolism	Others	unknown
2000	4	0	2	0	0	0	1	0	1	0
2001	7	2	3	0	1	0	1	0	0	0
2002	5	1	3	0	0	0	0	0	0	1
2003	5	1	2	1	0	0	0	0	1	0
2004	8	1	5	1	0	0	0	0	0	1
2005	4	0	1	0	1	1	0	0	0	1
2006	5	3	1	1	0	0	0	0	0	0
2007	0	0	0	0	0	0	0	0	0	0
2008	8	2	3	0	0	1	0	0	0	2
2009	9	2	4	1	1	0	0	0	0	1
2010	2	0	1	0	0	1	0	0	0	0
2011	5	3	1	0	1	0	0	0	0	0
2012	7	1	2	1	0	3	0	0	0	0
2013	2	0	2	0	0	0	0	0	0	0
2014	6	1	2	3	0	0	0	0	0	0
2015	6	2	1	1	0	0	0	1	0	1

© 2023 Cheung KW et al. *JAMA Network Open.*

Year	Late maternal death	Suicide	Cancer-related death	Cardiac diseases	Stroke	Infection (except HIV and hepatitis B)	HIV	Pulmonary embolism	Others	unknown
2016	2	0	0	1	1	0	0	0	0	0
2017	5	3	0	1	0	1	0	0	0	0
2018	6	0	5	0	0	0	0	0	1	0
2019	3	0	2	1	0	0	0	0	0	0
Total	99	22	40	12	5	7	2	1	3	7

© 2023 Cheung KW et al. *JAMA Network Open.*
