# Supplementary material for: Characteristics of Maternal Mortality Missed by Vital Statistics in Hong Kong, 2000-2019
Source: JAMA Netw Open. 2023 Feb 22;6(2):e230429. doi: 10.1001/jamanetworkopen.2023.0429 (PMC9947727; doi:10.1001/jamanetworkopen.2023.0429)
Supplement: Supplement 2. — Data Sharing Statement [file jamanetwopen-e230429-s002.pdf]

Data Sharing Statement

Cheung. Characteristics of Maternal Mortality Missed by Vital Statistics in Hong Kong, 2000-2019. *JAMA Netw Open*. Published February 22, 2023.
doi:10.1001/jamanetworkopen.2023.0429

Data
Data available: No

Additional Information
Explanation for why data not available: The data that support the findings of this study are available from the corresponding author upon reasonable request.
